# Experimental Performance of a Membrane Desorber with a H_2_O/LiCl Mixture for Absorption Chiller Applications

**DOI:** 10.3390/membranes12121184

**Published:** 2022-11-24

**Authors:** Jonathan Ibarra-Bahena, Ulises Dehesa-Carrasco, Yuridiana Rocio Galindo-Luna, Iván Leonardo Medina-Caballero, Wilfrido Rivera

**Affiliations:** 1 Subcoordinación de Conservación de Cuencas y Servicios Ambientales, Instituto Mexicano de Tecnología del Agua, Jiutepec 62550, Mexico; 2 Departamento de Ingeniería de Procesos e Hidráulica, Universidad Autónoma Metropolitana-Iztapalapa, Mexico City 09340, Mexico; 3 Instituto de Energías Renovables, Universidad Nacional Autónoma de México, Temixco 62580, Mexico

**Keywords:** absorption chiller, desorption, membrane distillation, LiCl mixture

## Abstract

For absorption cooling cycles using water as a refrigerant, H_2_O/LiCl mixtures are suitable for replacing conventional H_2_O/LiBr mixtures. In addition, membrane devices can be used to develop compact and lighter absorption systems, and they can operate with H_2_O/LiCl mixtures. The present paper describes an experimental evaluation of a membrane desorber/condenser operating at atmospheric pressure. Two operation modes were analyzed: continuous cycle operation and intermittent operation. For the first operation mode, the maximum desorption rate was 3.49 kg/h·m^2^, with a solution temperature of 90.3 °C and a condensation temperature of 25.1 °C. The lowest desorption rate value was 0.26 kg/h·m^2^, with a solution temperature of 75.4 °C and a condensation temperature of 40.1 °C. In the second mode, after three operating hours, the refrigerant fluid produced, per 1 m^2^ of membrane area, 7.7, 5.6, 4.3, and 2.2 kg, at solution temperatures of 90.3, 85.3, 80.4, and 75.4 °C, respectively. A one-dimension heat and mass transfer model is presented. The calculated values of desorption rate and outlet temperatures were compared with the experimental data; a square correlation coefficient of 0.9929 was reached for the desorption rate; meanwhile, for the outlet solution temperatures and the outlet cooling-water temperatures, a square correlation coefficient up to 0.9991 was achieved. The membrane desorber has the advantages of operating at atmospheric-pressure conditions, high condensation temperature, the ability to use different saline solution working mixtures, and different operation methods. These advantages can lead to new absorption systems.

## 1. Introduction

H_2_O/LiBr is the most used mixture in absorption chillers for air-conditioning applications. Water, as a refrigerant, shows convenient features: it has a high latent heat of evaporation; is a natural refrigerant; and is abundant, low cost, and harmless to the environment [1]. Absorption cycles using water as a refrigerant perform better than ammonia-based cycles, and a rectifier is unnecessary for them [2]. Among the water-based mixtures, H_2_O/LiCl mixtures are an interesting alternative showing some advantages compared with H_2_O/LiBr mixtures: triple-state point (solid, liquid, and vapor forms), long-term stability, and lower cost [3]. In addition, H_2_O/LiCl mixtures show better hygroscopic properties than other aqueous solutions, such as H_2_O/CaCl_2_ [4], although the smaller chemical potential of LiCl relative to LiBr leads to a larger absorber area [5]. On the other hand, Ahamad et al. [6], through a simulation analysis, found that at similar operating system temperatures, the solution concentrations in the desorber and absorber were considerably lower for the H_2_O/LiCl mixture than for the H_2_O/LiBr mixture, considerably reducing the risk of crystallization in these components. Additionally, they found that a slightly higher maximum Coefficient Of Performance (COP) could be reached with the H_2_O/LiCl mixture. Furthermore, the desorber temperature is lower with H_2_O/LiCl mixtures because the vapor pressure is higher than that in conventional H_2_O/LiBr mixtures [7]. The convenience of H_2_O/LiCl mixtures has already been demonstrated: higher COP [8,9], higher exergy efficiency [10,11], and a high potential to be integrated into thermal solar energy systems [12,13].

Additionally, using the correct working mixture allows the development of compact components for small-scale duties, such as single-family houses and automotive applications [14,15]. Light and small devices must replace conventional heat exchangers used as desorbers. In this regard, membrane modules constitute a novel technology that provides a high heat and mass transfer rate with a reduced size [16]. Studies on using aqueous lithium chloride mixtures with membrane components for absorption chiller systems are scarce: De Vega et al. [17] used microchannel membrane absorber simulation analysis to develop compact absorption chillers. The authors analyzed H_2_O/LiBr, NH_3_/LiNO_3_, and H_2_O/LiCl mixtures, and a membrane absorber with 13 microchannels 0.15 mm in height, 1.5 mm in width, and 50 mm in length was simulated. A membrane with 60 μm thickness, 80% porosity, and a pore diameter of 1 μm was assumed. According to the authors’ results, the highest ratio between cooling capacity and absorber volume was reached with the NH_3_/LiNO_3_ mixture. The H_2_O/LiCl mixture had the lowest desorption temperature; however, the COP was also the lowest. Asfand et al. [18] carried out a 2D simulation of a plate-and-frame membrane absorber that operates with H_2_O/(LiBr + LiI + LiNO_3_ + LiCl) and H_2_O/(LiNO_3_ + KNO_3_ + NaNO_3_) mixtures. The membrane absorber was 0.5 mm in width and 200 mm in length, and a membrane with 40 μm thickness, 85% porosity, and a pore diameter of 1 μm was considered. According to the authors’ results, the absorption rate increased by 25% with the H_2_O/(LiBr + LiI + LiNO_3_ + LiCl) mixture compared with H_2_O/LiBr under air-cooling thermal conditions. In addition, an absorption rate of 0.00523 kg/s × m^2^ was reached when the H_2_O/(LiNO_3_ + KNO_3_ + NaNO_3_) mixture was used in the membrane-based absorber of the third stage of a triple-effect absorption cooling cycle.

In addition, there are several studies on membrane absorption heat pumps using H_2_O/LiCl mixtures. These devices can be used for air-dehumidification, water-heating, and absorption cooling applications. Yang et al. [19] carried out a 3D simulation on a counter-flow parallel-plate membrane absorption heat pump (PMAHP) to recover low-temperature waste heat from used cooling water at 40 °C with a H_2_O/LiCl mixture. The authors concluded that the optimum operating conditions for the PMAHP were as follows: lower solution flow rate, higher water flow rate, lower inlet solution mass fraction, and higher inlet water temperature. Huang [20] analyzed the heat and mass transfer in a cross-flow hollow-fiber membrane absorption heat pump (HFMAHP) using a H_2_O-LiCl mixture. The solution temperature lift and the overall heat and mass transfer coefficients increased as the skin layer thickness increased. According to the author, the skin layer effect was higher than the porous layer. In another paper, Huang [21] analyzed a quasi-counter-flow parallel-plate membrane-absorption heat pump (QPMAHP) for fluid heating with a H_2_O/LiCl mixture. The author concluded that the sensible and latent heat transfer across the membranes was due to the large overall heat and mass transfer resistances. Nonetheless, the latent heat flux was dominant. The solution temperature lift and efficiency increased until it reached 9.1%, when the entrance and aspect ratios were equal to 0.1. Woods et al. [22] designed and tested a membrane absorption heat pump. The system included a membrane absorber with two hollow-fiber rows; the aqueous salt solution flowed inside one row, whereas the water flowed in the other. H_2_O/CaCl_2_ and H_2_O/LiCl mixtures were used. The authors mentioned that the cost of LiCl was higher than that of CaCl_2_. Performance comparison of a hollow-fiber membrane module with Oxyphan membranes and Accurel membranes was carried out assuming that they had the same membrane physical characteristics. The authors found that the mass transfer coefficient with Oxyphan membranes was 4.2 times higher than that with the second type. The water vapor mass flux was 2.1 times higher with the Accurel membranes compared with the Oxyphan ones.

The literature reviewed demonstrates the suitability of H_2_O/LiCl mixtures with membrane devices, especially for absorption cooling applications. The aim of the present study was to demonstrate the suitability of the H_2_O/LiCl mixture for the operation of a membrane desorber for absorption-cooling-system applications. This paper presents an experimental evaluation of a membrane desorber with a H_2_O/LiCl mixture at atmospheric-pressure conditions. The membrane device uses the air gap membrane distillation (AGMD) configuration. Two operation modes were analyzed: (i) continuous cycle operation and (ii) intermittent operation. Furthermore, the effects of the solution temperature and cooling-water temperature on the desorption rate were analyzed, and a one-dimension heat and mass transfer model was constructed.

## 2. Air Gap Membrane Distillation (AGMD) Configuration

**Figure 1:**
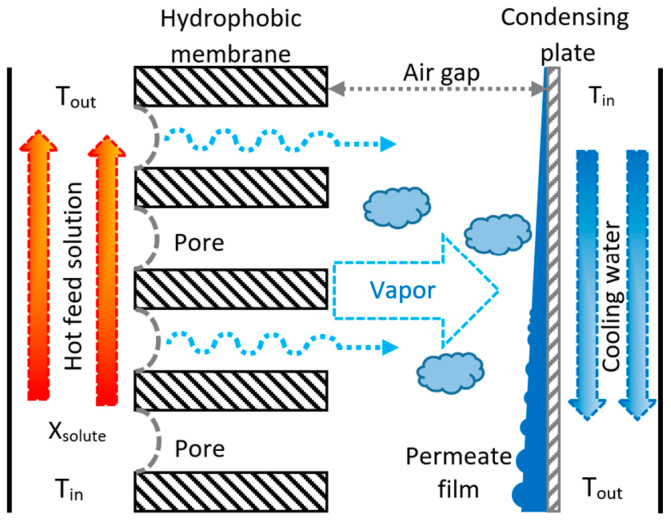
Schematic diagram of the AGMD separation process [25].

Membrane distillation is a thermal separation process using a hydrophobic membrane as an interphase contactor between two fluids at different temperatures, where the separation occurs by the vapor mass transfer. At relatively low operating pressures, the hydrophobicity of the membrane prevents the liquid phase from wetting the membrane pores, and vapor is the only phase to cross the membrane. The temperature difference between the two sides of the membrane produces the driving force for the vapor mass transfer from the fluid at a higher temperature (hot side) to the one at a lower temperature (cold side) [23]. A schematic diagram of the AGMD configuration is shown in Figure 1. Some of the advantages of the AGMD configuration are as follows: the air gap serves as a thermal insulation layer and reduces the heat loss from the membrane; latent heat is recovered without an external heat exchanger; thermal efficiency is higher; it is suitable for pilot testing plants; and the AGMD modules can be scaled up and made with polymeric corrosion-resistant materials [24].

## 3. Methodology

The membrane desorber/condenser assessment was performed by varying the main system’s operating parameters, such as LiCl mass flow rate (*ṁ_LiCl_*), inlet solution temperature (*T_LiCl,in_*), and inlet cooling-water temperature (*T_cw,in_*). Additionally, two operation modes were analyzed. In the first, a continuous operation absorption cycle was assumed, which means that the initial LiCl concentration was constant. In the second case, intermittent operation mode was assumed, meaning that the desorption/condensation and the evaporation/absorption processes in an absorption system separately occurred and that the LiCl concentration increased with operation time.

### 3.1. Experimental Setup

**Figure 2:**
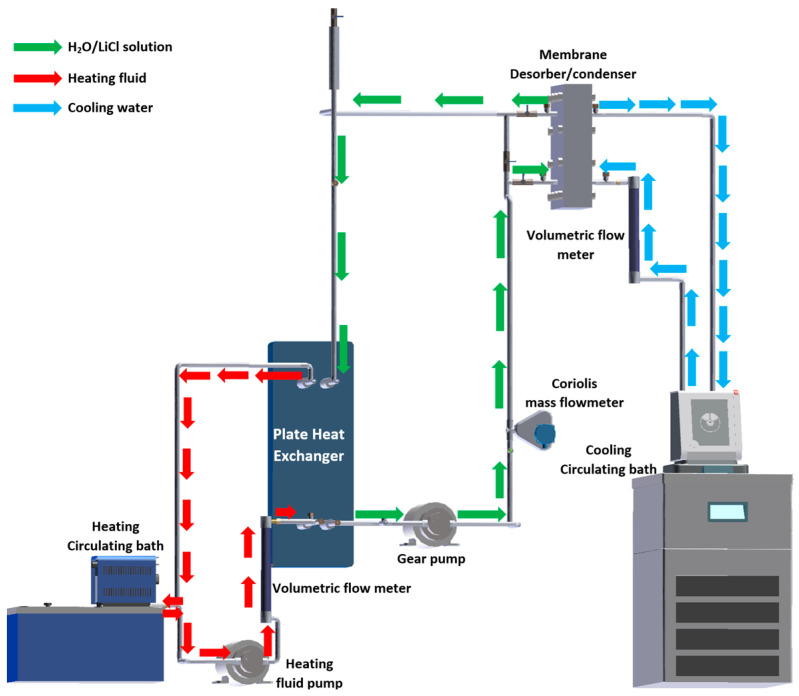
Schematic diagram of the experimental setup.

**Figure 3:**
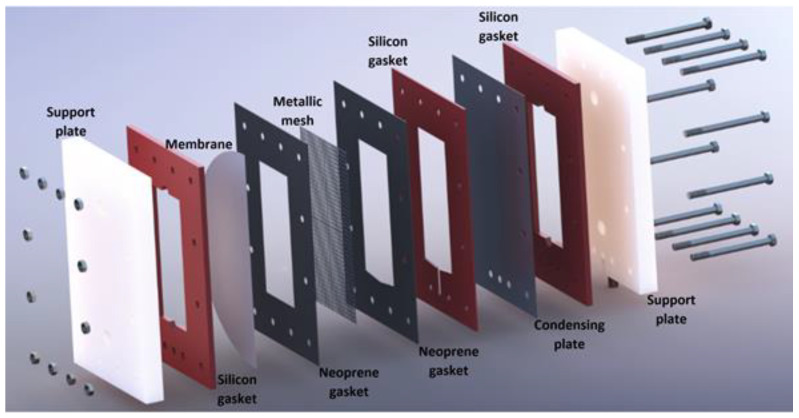
Experimental membrane desorber/condenser unit [25].

The experimental setup was mainly integrated by a membrane desorber/condenser unit, heating system, and cooling system, as shown in Figure 2. The membrane desorber/condenser unit functioned to separate part of the water contained in the LiCl solution as previously described. The unit comprised two Nylamid support plates, neoprene gaskets, a metallic mesh, an aluminum cooling plate, and a PTFE membrane, as shown in Figure 3.

The characteristics of the hydrophobic membrane are shown in Table 1.

The heating system comprises a heating bath with temperature control, a stainless-steel plate heat exchanger (PHE), a pump, and a Coriolis mass flowmeter. This system is in charge of supplying the necessary heat to the LiCl solution in the PHE before the solution enters the desorber unit.

The cooling system comprises a circulating cooling bath with temperature control and an integrated pump. This system provides the cooling to the desorber/condenser unit to condense the water in a vapor phase passing through the membrane.

**Figure 4:**
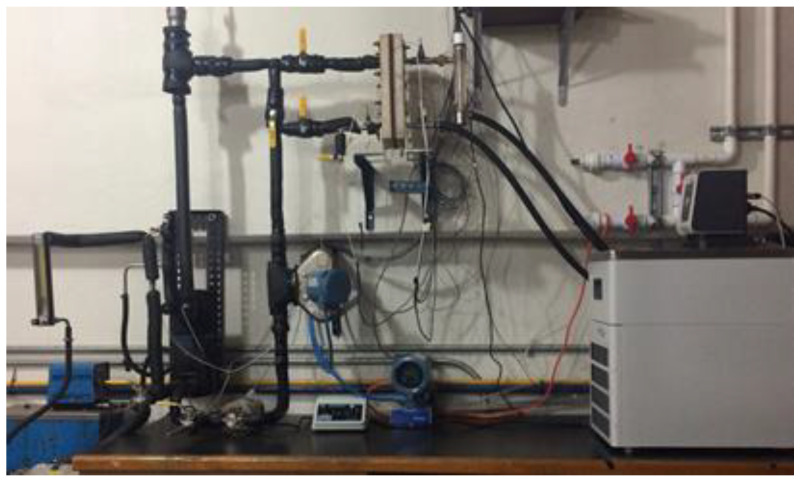
Experimental setup [25].

A detailed description of the membrane desorber/condenser unit was already reported by Ibarra et al. [25]. Figure 4 shows a photograph of the experimental setup.

For the continuous operation mode, 16 experimental test runs were carried out; meanwhile, 4 test runs in intermittent operation mode were completed. The operating temperatures were selected on the basis of cooling absorption systems operating with renewable thermal energies [12,13] or low-grade heat sources [26]. The uncertainties of the measured variables and instruments used in the experimental test runs are shown in Table 2, and Table 3 and Table 4 report the experimental operating conditions for each operation mode.

### 3.2. Heat and Mass Transfer Model

**Figure 5:**
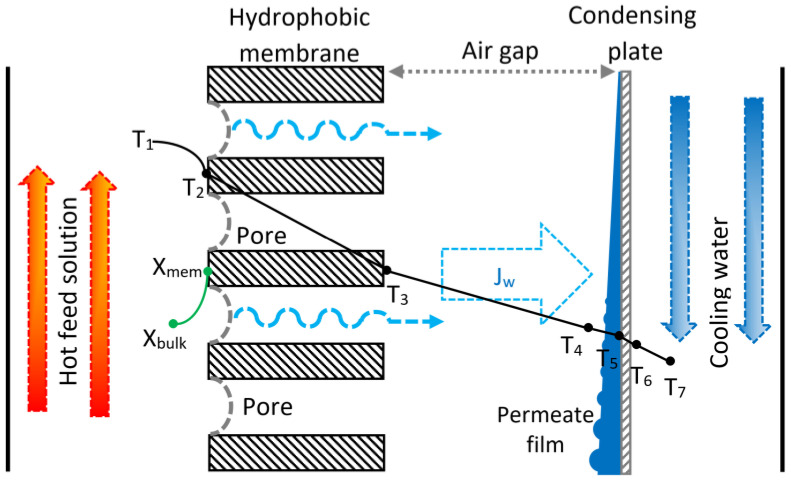
Conceptual diagram of the AGMD process.

A conceptual diagram of the desorber/condenser unit is shown in Figure 5. The hot saline solution is in direct contact with one side of the porous membrane. The refrigerant fluid evaporates at the entrance of the pores because of the hydrophobic nature of the membrane while a concentration profile is created: the salt concentration of the bulk fluid (*X_bulk_*) increases until a higher concentration at the membrane interface (*X_mem_*). The working fluid phase change occurs according to the vapor–liquid equilibrium of the H_2_O/LiCl mixture. The vapor permeates the membrane through the pores by the Poiseuille–Knudsen mechanism, crosses the air gap by diffusion, and condenses on the refrigerated plate. The heat transfer through the system is driven by the temperature difference between the hot saline solution channel and the cooling-water channel. The vapor mass transfer simultaneously occurs in the same direction with the heat transfer. The coupling of these phenomena is called the Soret effect.

In order to estimate the global performance parameters to scale and design a membrane desorber/condenser, a previously described one-dimension heat and mass transfer model was used [27] and adapted; it includes the H_2_O/LiCl mixture equilibrium conditions to describe the vapor water desorption. The following assumptions were made:

(1) The desorber/condenser unit operates at steady state conditions.
(2) Thermophysical properties are constant.
(3) The heat and mass transfer processes occur in one dimension.
(4) Natural convection is neglected in the air gap region.
(5) Liquid–vapor equilibrium exists at the evaporation and condensation interfaces.

Energy conservation was considered in the different regions inside the experimental device [28,29].

The desorption heat flux (*Q_D_*) transferred from the hot saline solution to the membrane is given by:

(1) $$Q_{D}=h_{1}\left(T_{1}-T_{2}\right)$$

The heat transferred to the membrane:

(2) $$h_{1}\left(T_{1}-T_{2}\right)=J_{w}\lambda_{w}+\frac{k_{mem}}{\delta_{mem}}\left(T_{2}-T_{3}\right)$$

Inside the air gap:

(3) $$h_{1}\left(T_{1}-T_{2}\right)=J_{w}\lambda_{w}+\frac{k_{gap}}{\delta_{gap}}\left(T_{3}-T_{4}\right)$$

In the permeate film:

(4) $$h_{1}\left(T_{1}-T_{2}\right)=h_{4}\left(T_{4}-T_{5}\right)$$

At the condensing plate:

(5) $$h_{1}\left(T_{1}-T_{2}\right)=\frac{k_{p}}{\delta_{p}}\left(T_{5}-T_{6}\right)$$

In the cooling stream:

(6) $$h_{1}\left(T_{1}-T_{2}\right)=h_{6}\left(T_{6}-T_{7}\right)$$

where *h* is the convective heat transfer coefficient, *J_w_* is the desorption rate, *λ_w_* is the latent heat of vaporization of water, *k* is the thermal conductivity, and *δ* refers to the thickness. Subscripts *mem*, *gap*, and *p* refer to the membrane, air gap, and condensation plate, respectively. Subscripts 1 to 7 are the numerical notation in Figure 5.

*Q_D_* can be simplified in a mathematical expression as a function of the H_2_O/LiCl mixture temperature and the cooling-water stream temperature (*T*_1_ and *T*_7_, respectively). The equation system, integrated by Equations (2)–(6), was solved to dismiss the intermediate temperatures (*T*_2_ to *T*_6_). Thus, Equation (7) is proposed:

(7) $$Q_{D}=\frac{\psi \omega }{\psi +k_{eq}}\left[N_{A}\lambda_{A}+k_{eq}\left(T_{1}-T_{7}\right)\right]$$

where the *ψ*, *ω*, *k_eq_*, and *h_eq_* are defined, respectively, as follows:

(8) $$\psi =\frac{h_{6}k_{p}}{\delta_{p}h_{6}+k_{p}}$$

(9) $$\omega =\frac{h_{eq}}{k_{eq}+h_{eq}}$$

(10) $$k_{eq}=\frac{k_{mem}k_{gap}}{\delta_{gap}k_{mem}+\delta_{mem}k_{gap}}$$

(11) $$h_{eq}=\frac{h_{1}h_{4}}{h_{1}+h_{4}}$$

The convective heat transfer coefficients *h*_1_ and *h*_6_ were estimated from empirical correlations. In the H_2_O/LiCl mixture channel, the correlation proposed by Shah and London [30] was used to calculate the Nusselt number, which is valid for a fully developed flow in thin rectangular channels. On the other hand, in the cooling-water channel, the correlation reported by Khayet [31] was used. The correlation proposed by Bird et al. [32] was used to calculate the convective heat transfer coefficient for the condensate film (*h*_4_).

The temperatures for each zone inside the desorber/condenser device (*T*_2_ to *T*_6_) were calculated from Equations (1)–(6). However, *T*_1_ and *T*_7_, considered as the bulk temperatures of H_2_O/LiCl solution and cooling-water stream, respectively, were assumed to be the experimental inlet and outlet temperatures, respectively. Stream inlet temperatures (*T_LiCl,in_* and *T_cw,in_*) are input parameters for the mathematical model, and the outlet temperatures were calculated by a numerical procedure, as a function of the heat flux in both streams:

(12) $$T_{LiCl,out}=T_{LiCl,in}-\frac{Q_{D}+Q_{l}}{C_{p,LiCl}\;{\dot{m}}_{LiCl}}$$

(13) $$T_{cw,out}=T_{cw,in}-\frac{Q_{D}+Q_{l}}{C_{p,cw}\;{\dot{m}}_{cw}}$$

where *Cp* is the heat capacity, *ṁ* is the mass flow rate, and *Q_l_* is the heat loss to the environment.

The ratio between the mass fraction at the liquid–membrane interface (*X_m_*) and the mass fraction in the bulk flow (*X_b_*) is defined as follows [22,33]:

(14) $$\frac{X_{mem}}{X_{b}}=\exp\left(\frac{J_{w}}{\varphi_{bl}*\rho_{LiCl}}\right)$$

where *φ_bl_* is the convective mass transfer coefficient at the boundary layer, which was calculated by the correlation proposed by Woods et al. [33]; and *ρ_LiCl_* is the H_2_O/LiCl solution density.

The driving force of the desorption rate (*J_w_*) is the partial pressure difference between the interface de evaporation (*p*_1_) and condensate film (*p*_4_). However, the mass transfer flux is restricted by a global mass transfer coefficient (*K_ov_*):

(15) $$J_{w}=K_{ov}\left(p_{1}-p_{4}\right)$$

The global mass transfer coefficient involves the boundary layer mass transfer coefficient at the liquid–membrane interface (*K_bl_*), the mass transfer coefficient on the membrane (*K_mem_*), and the mass transfer coefficient for the air gap (*K_gap_*). It is expressed as:

(16) $$K_{ov}={\left[\frac{1}{K_{bl}}+\frac{1}{K_{mem}}+\frac{1}{K_{gap}}\right]}^{-1}$$

The mass transfer coefficient for the membrane is expressed as:

(17) Kmem=MwRTmemδmem[(23rpετ{8RTmemπMw}12)−1+(DvapτPplm)−1]−1

The mass transfer coefficient for the air gap is defined as:

(18) $$K_{gap}=\frac{M_{w}}{RT_{gap}}\frac{D_{vap}}{\delta_{gap}}\frac{P}{p_{lm}}$$

The boundary layer mass transfer coefficient is defined as:

(19) $$K_{bl}=\frac{J_{w}}{p_{b}-p_{bl}}$$

In these equations, *M_w_* is the molecular mass of water; *R* is the universal gas constant; *P* and *p_lm_* are the total operating pressure and the logarithmic-mean pressure of air, respectively; *δ_mem_*, *ε*, and *τ* are the thickness, porosity, and tortuosity of the membrane, respectively; *D_vap_* is the water vapor/air binary mass diffusion coefficient; *r_p_* is the membrane mean pore radius; *δ_gap_* is the air gap thickness; and *p_b_* and *p_bl_* are the vapor partial pressure at the bulk layer and the boundary layer, respectively. The thermodynamic properties of the H_2_O/LiCl solution were calculated by using the correlations reported by Chaudhari and Patel [34] and Wimby and Berntsson [35].

In the experimental device, the channel length is small; therefore, the temperature difference between the inlet and the outlet is smaller than the temperature difference across the hot saline solution channel and cooling-water channel. Thus, a one-dimensional model assumption allows a general description of the system for engineering design purposes.

## 4. Results

### 4.1. Continuous Cycle Operation

**Figure 6:**
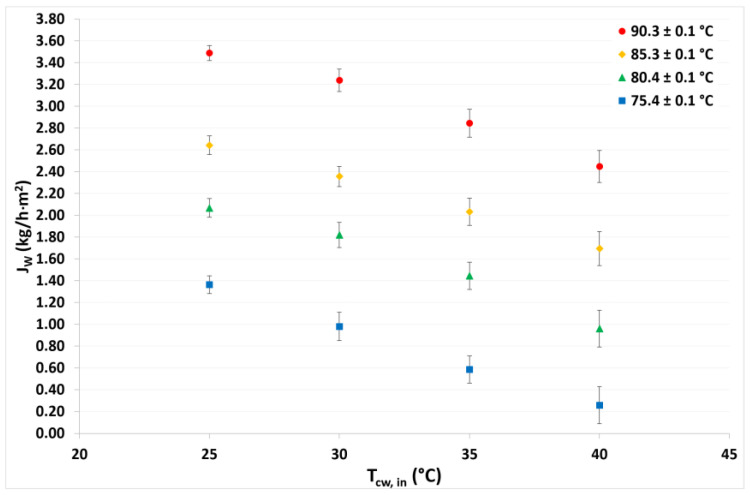
Desorption rate as a function of the solution and condensation temperatures.

The experimental desorption rate (*J_w_*) was measured on the basis of the mass of distillate water produced by the membrane desorber after a defined time period, under different operating conditions. As demonstrated in previous reports with LiBr aqueous solutions [25,27], the desorption rate is mainly affected by the solution temperature rather than by the condensation temperature. For instance, at a solution temperature of 90.3 °C at the lowest condensation temperature, the desorption rate was 2.6 times higher than at 75.4 °C for the same condensation temperature. Meanwhile, the desorption rate at the highest condensation temperature was 9.4 times higher at the solution temperature of 90.3 °C than at 75.4 °C. As can be seen in Figure 6, the influence of the condenser temperature in the desorption rate was greater at the lowest solution temperature because at 75.4 °C the desorption rate was 5.2 times higher at the lowest condenser temperature than at the highest temperature; meanwhile, at the solution temperature of 90.3 °C, the desorption rate was just 1.4 times higher at the same condenser temperatures. The maximum desorption rate was 3.49 kg/h·m^2^ at a solution temperature of 90.3 °C and a condensation temperature of 25.1 °C. On the other hand, the lowest desorption rate value was 0.26 kg/h·m^2^ at a solution temperature of 75.4 °C and a condensation temperature of 40.1 °C. Because the vapor mass transfer driving force depends on the temperature difference between both sides of the membrane, as this temperature difference increases, the desorption rate also increases.

In Table 5, a comparison of the experimental results of this work with what was reported in the literature is presented. The desorption rates with the H_2_O/LiCl mixture are slightly lower than those reported with the H_2_O/LiBr mixture under similar operating conditions and with the same membrane configuration; however, the desorption rates are in a similar range to those reported with the H_2_O/LiBr mixture. These results show the suitability of using the H_2_O/LiCl mixture in a desorption process with membrane devices for absorption cooling systems.

### 4.2. Intermittent Operation

**Figure 7:**
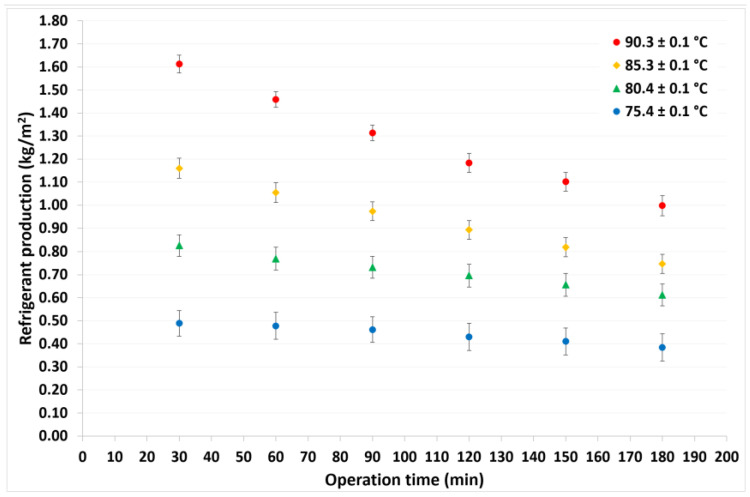
Refrigerant production as a function of operation time.

**Figure 8:**
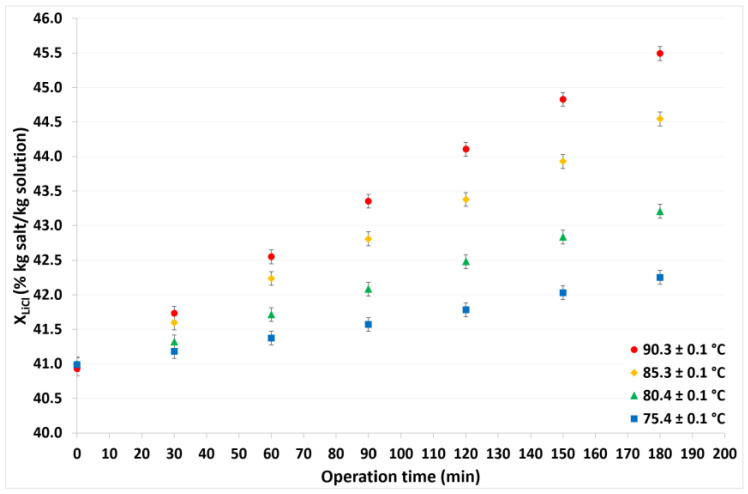
LiCl concentration as a function of operation time.

For this analysis, the refrigerant production (or water distilled) was defined as the quantity, in kilograms, of the refrigerant fluid produced per 1 m^2^ of membrane area. After three operating hours, at a constant condensation temperature of 30.1 °C, the refrigerant fluid produced was 7.7, 5.6, 4.3, and 2.2 kg, at solution temperatures of 90.3, 85.3, 80.4, and 75.4 °C, respectively. Figure 7 shows that the refrigerant produced decreased with operation time and showed a nonlinear dependence, particularly with high solution temperatures. As previously mentioned, the mass transfer driving force increases at higher temperature differences; thus, the refrigerant production was higher at the highest solution temperature. On the other hand, as a function of operation time, the amount of refrigerant contained in the solution decreased and the LiCl concentration increased, as is shown in Figure 8, so the refrigerant production decreased. Moreover, this behavior was observed in a previous study [46].

### 4.3. Model Validation

**Figure 9:**
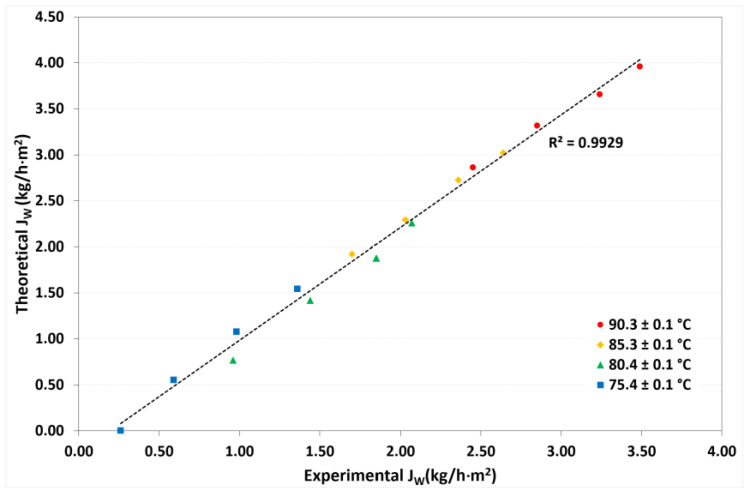
Comparison between theoretical and experimental desorption rates.

**Figure 10:**
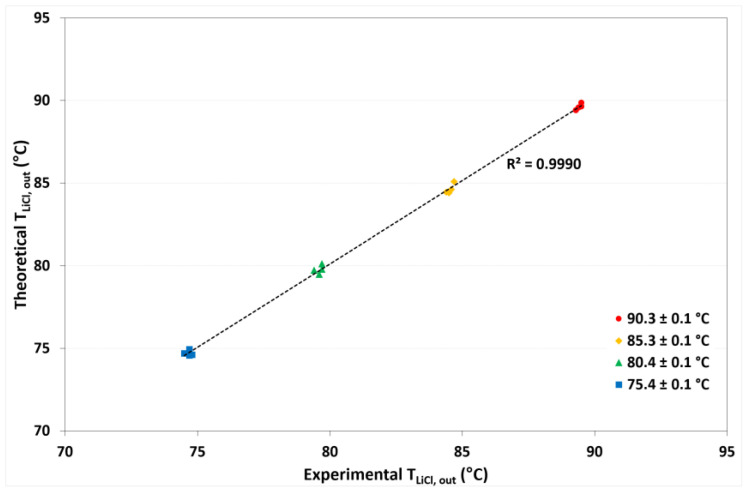
Comparison between theoretical and experimental outlet solution temperatures.

**Figure 11:**
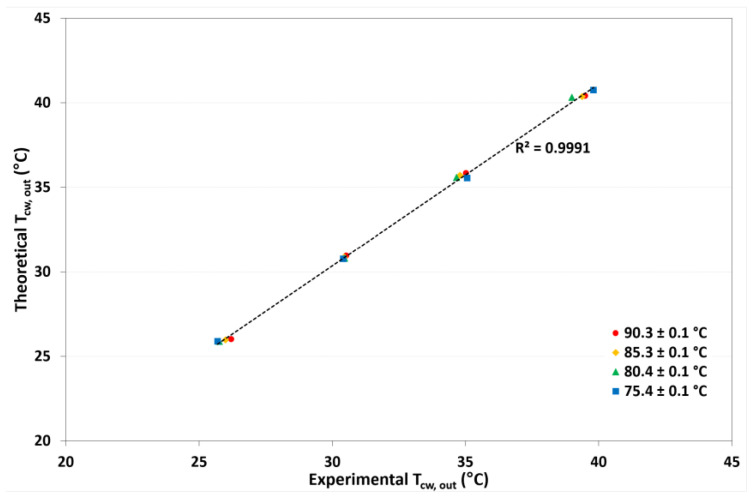
Comparison between theoretical and experimental outlet cooling temperatures.

On the basis of the one-dimensional heat and mass transfer model, the desorption rate (*J_W_*), outlet solution temperatures (*T_LiCL,out_*), and outlet cooling-water temperatures (*T_cw,out_*) were calculated. Figure 9 shows the comparison of theoretical and experimental refrigerant desorption rates. A square correlation coefficient (R^2^) of 0.9929 was reached, which means that the mathematical model provided values close to the experimental data. Figure 10 and Figure 11 show the theoretical *T_LiCl,out_* as a function of the experimental *T_LiCl,out_* and the theoretical *T_cw,out_* as a function of the experimental *T_cw,out_*, respectively. In both cases, an R^2^ value up to 0.9991 was achieved. This validation is essential for design heat networks to improve the heat efficiency of the absorption chillers.

One way to improve the mathematical model is to divide the channels into vertical control volumes, where each one has a uniform temperature. This means considering several vertical one-dimensional heat transfer paths in parallel.

## 5. Conclusions

A membrane desorber/condenser unit was experimentally evaluated, under laboratory conditions, with a H_2_O/LiCl solution. Two operation modes were analyzed: continuous cycle operation and intermittent operation. In the first case, four solution temperatures (90.3, 85.3, 80.4, and 75.4 °C) and four condensation temperatures (25.1, 30.1, 35.1, and 40.1 °C) were evaluated. According to the results, the desorption rate was principally affected by the solution temperature rather than by the condensation temperature; however, the condensation temperature effect on the desorption rate was notable at the lowest solution temperature. The maximum desorption rate was 3.49 kg/h·m^2^ at a solution temperature of 90.3 °C and condensation temperature of 25.1 °C.

On the other hand, the lowest desorption rate value was 0.26 kg/h·m^2^ at a solution temperature of 75.4 °C and condensation temperature of 40.1 °C. The same four solution temperatures as the first operation case were analyzed in the second mode at a constant condensation temperature of 30.1 °C. Mathematical model validation was carried out, and a square correlation coefficient of 0.9929 was reached for the desorption rate; meanwhile, for the outlet solution temperatures and the outlet cooling-water temperatures, a square correlation coefficient up to 0.9991 was achieved. After three operating hours, the refrigerant fluid produced, assuming a 1 m^2^ of membrane area, was 7.7, 5.6, 4.3, and 2.2 kg with solution temperatures of 90.3, 85.3, 80.4, and 75.4 °C, respectively. The refrigerant production increased as the solution temperature increased; however, the refrigerant production decreased with operation time because the LiCl concentration increased. On the basis of the experimental results, the H_2_O/LiCl mixture can be used to replace the conventional H_2_O/LiBr mixture for absorption cooling systems that integrate membrane devices.

## Figures and Tables

**Table 1:** Membrane characteristics.

Material	PTFE (Polytetrafluoroethylene)
Mean pore diameter (*d_p_*)	0.22 μm
Porosity (*ε*)	70%
Effective area (*A_mem_*)	144 cm^2^
Thickness (*δ_mem_*)	175 μm

**Table 2:** Uncertainty of the measured variables.

Variable	Sensor/Instrument	Operation Range	Uncertainty
Temperature	RTD PT100	−30 to 350 °C	±0.1 °C
Volumetric flow	Volumetric flowmeter	0 to 7 L/min	±5.0% f.s. *
Mass flow	Coriolis mass flowmeter	0 to 4.0 × 10^−2^ kg/s	±0.1%
Distillate water weight	Electronic balance	0 to 600 g	±0.01 g

* f.s., full scale.

**Table 3:** Experimental operating conditions in continuous operation mode.

Parameter	Value
LiCl concentration (% kg salt/kg solution)	41.05 ± 0.03
Cooling-water volumetric flow (L/min)	2.0 ± 0.35
H_2_O/LiCl solution mass flow (kg/s)	3.50 × 10^−2^ ± 1.83 × 10^−5^
H_2_O/LiCl solution temperature (°C)	90.3 ± 0.1
	85.3 ± 0.1
	80.4 ± 0.1
	75.4 ± 0.1
Cooling-water temperature (°C)	40.1 ± 0.1
	35.1 ± 0.1
	30.1 ± 0.1
	25.1 ± 0.1

**Table 4:** Experimental operating conditions in intermittent operation mode.

Parameter	Value
LiCl concentration (% kg salt/kg solution)	40.98 ± 0.03
Cooling-water volumetric flow (L/min)	2.0 ± 0.35
H_2_O/LiCl solution mass flow (kg/s)	3.00 × 10^−2^ ± 4.90 × 10^−5^
H_2_O/LiCl solution temperature (°C)	90.3 ± 0.1
	85.3 ± 0.1
	80.4 ± 0.1
	75.4 ± 0.1
Cooling-water temperature (°C)	30.1 ± 0.1

**Table 5:** Comparison of the experimental desorption rates for membrane desorbers reported in the literature.

Reference	Configuration	*d_p_* (μm)	Working Mixture	*X* (% *w*/*w*) *	*T_sol_* (°C)	*T_Con_* (°C)	*ṁ_sol_* (kg/h)	*J_w_* (kg/h·m^2^)
[36]	Flat sheet	1.0	H_2_O/LiBr	50 to 60	65 to 85	40	15 to 50	14 to 72
[37]	Flat sheet	0.45	H_2_O/LiBr	43	70 to 90	37 to 47	2.4 to 3.6	7.2 to 14.4
[38]	Hollow fiber	0.2 to 0.4	H_2_O/LiBr	58	90	40	10 to 30	0.41 to 2.2
[25]	Flat sheet	0.22	H_2_O/LiBr	49.6	80 to 95	30 to 45	90 to 144	1.1 to 6.1
[39]	Flat sheet	0.45	H_2_O/LiBr	45.8	58 to 60	25.7	0.5 to 1.7	5.8 to 15.1
[40]	Flat sheet	0.22	H_2_O/LiBr	49.8	75.2 to 95.3	14.4 to 25.4	90.0	1.5 to 5.7
[41]	Hollow fiber	0.16	H_2_O/LiBr	51 to 58	65 to 83	NA	173 to 269	0.4 to 3.4
[27]	Flat sheet	0.45	H_2_O/LiBr	45.7 to 58.7	74.4 to 95.9	15.6 to 20.0	58.7 to 90.0	0.3 to 9.7
[42]	Flat sheet	0.45	H_2_O/LiBr	48 to 51	50 to 125	NA	0.75 to 3.25	0.0 to 37.8
[43]	Flat sheet	1.00	H_2_O/LiBr	48	50 to 125	NA	2.5	0.0 to 34.2
[44]	Hollow fiber	0.16	H_2_O/LiBr	50	65 to 88	NA	40 to 120	0.3 to 2.0
[45]	Flat sheet	0.20	H_2_O/LiBr	35 to 55	35 to 100	15	NA	1.8 to 18
This work	Flat sheet	0.22	H_2_O/LiCl	41	75 to 90	25 to 40	126	0.26 to 3.49

* kg salt/kg solution.

## Data Availability

The data presented in this study are available on request from the corresponding.

## References

[1] Arshi Banu P.S., Sudharsan N.M. (2017). Review of water based vapour absorption cooling systems using thermodynamic analysis. Renew. Sustain. Energy Rev..

[2] Jian S., Lin F., Zhang S. (2012). A review of working fluids of absorption cycles. Renew. Sustain. Energy Rev..

[3] Altamirano A., Le Pierrès N., Stutz B. (2019). Review of small-capacity single-stage continuous absorption systems operating on binary working fluids for cooling: Theoretical, experimental and commercial cycles. Int. J. Refrig..

[4] Gommed K., Grossman G., Ziegler F. (2004). Experimental investigation of a LiCI-water open absorption system for cooling and dehumidification. J. Sol. Energy Eng..

[5] Kim K., Ameel T., Wood B. (1997). Performance evaluations of LiCl and LiBr for ab- sorber design applications in the open-cycle absorption refrigeration system. J. Sol. Energy Eng..

[6] Ahmad T., Azhar M., Sinha M.K., Meraj M., Mahbubul I.M., Ahmad A. (2022). Energy analysis of lithium bromide-water and lithium chloride-water based single effect vapour absorption refrigeration system: A comparison study. Clean. Eng. Technol..

[7] Saravanan R., Maiya M.P. (1998). Thermodynamic comparison of water-based working fluid combinations for a vapour absorption refrigeration system. Appl. Therm. Eng..

[8] Horuz I. (1998). A comparison between ammonia-water and water-lithium bromide solutions in vapor absorption refrigeration systems. Int. Commun. Heat Mass Transf..

[9] Barragán Reyes R.M., Arellano Gómez V.M., García-Gutiérrez A. (2010). Performance modelling of single and double absorption heat transformers. Curr. Appl. Phys..

[10] Patel J., Pandya B., Mudgal A. (2017). Exergy based analysis of LiCl-H_2_O absorption cooling system. Energy Procedia.

[11] Bhowmick A., Kundu B. (2021). Thermo-economic optimization and comparison study of LiBr-H_2_O and LiCl-H_2_O working pair in absorption cooling systems based on genetic algorithm. Int. J. Energy Res..

[12] Bellos E., Tzivanidis C., Antonopoulos K.A. (2016). Exergetic and energetic comparison of LiCl-H_2_O and LiBr-H_2_O working pairs in a solar absorption cooling system. Energy Convers. Manag..

[13] Bellos E., Tzivanidis C., Pavlovic S., Stefanovic V. (2017). Thermodynamic investigation of LiCl-H_2_O working pair in a double effect absorption chiller driven by parabolic trough collectors. Therm. Sci. Eng. Prog..

[14] Labus J.M., Bruno J.C., Coronas A. (2013). Review on absorption technology with emphasis on small capacity absorption machines. Therm. Sci..

[15] Altamirano A., Stutz B., Le Pierrès N. (2020). Review of small-capacity single-stage continuous absorption systems operating on binary working fluids for cooling: Compact exchanger technologies. Int. J. Refrig..

[16] Yu D., Chung J., Moghaddam S. (2012). Parametric study of water vapor absorption into a constrained thin film of lithium bromide solution. Int. J. Heat Mass Transf..

[17] De Vega M., Venegas M., García-Hernando N. (2018). Modeling and performance analysis of an absorption chiller with a microchannel membrane-based absorber using LiBr-H_2_O, LiCl-H_2_O, and LiNO_3_-NH_3_. Int. J. Energy Res..

[18] Asfand F., Stiriba Y., Bourouis M. (2016). Performance evaluation of membrane-based absorbers employing H_2_O/(LiBr + LiI + LiNO_3_ + LiCl) and H_2_O/(LiNO_3_ + KNO_3_ + NaNO_3_) as working pairs in absorption cooling systems. Energy.

[19] Yang M., Low E., Lim Law C., Chen J.-C., Show P.L., Huang S.-M. (2022). Heat and mass transfer in a counter flow parallel plate membrane-based absorption heat pump (PMAHP). Int. J. Therm. Sci..

[20] Huang S.-M. (2017). Coupled heat and mass transfer in a cross-flow hollow fiber membrane absorption heat pump (HFMAHP). Appl. Therm. Eng..

[21] Huang S.M. (2015). Heat and mass transfer in a quasi-counter flow parallel-plate membrane-based absorption heat pump (QPMAHP). J. Membr. Sci..

[22] Woods J., Pellegrino J., Kozubal E., Burch J. (2011). Design and experimental characterization of a membrane-based absorption heat pump. J. Membr. Sci..

[23] Alsaadi A.S., Ghaffour N., Li J.-D., Gray S., Francis L., Maab H., Amy G.L. (2013). Modeling of air gap membrane distillation process: A theoretical and experimental study. J. Membr. Sci..

[24] Shahu V.T., Thombre S.B. (2019). Air gap membrane distillation: A review. J. Renew. Sustain. Energy.

[25] Ibarra-Bahena J., Rivera W., Nanco-Mejía S.D., Romero R.J., Venegas-Reyes E., Dehesa-Carrasco U. (2021). Experimental performance of a membrane desorber operating under simulated warm weather condensation temperatures. Membranes.

[26] She X., Yin Y., Xu M., Zhang X. (2015). A novel low-grade heat-driven absorption refrigeration system with LiCl–H_2_O and LiBr–H_2_O working pairs. Int. J. Refrig..

[27] Ibarra-Bahena J., Dehesa-Carrasco U., Romero R.J., Rivas-Herrera B., Rivera W. (2017). Experimental assessment of a hydrophobic membrane-based desorber/condenser with H_2_O/LiBr mixture for absorption systems. Exp. Therm. Fluid Sci..

[28] Izquierdo-Gil M.A., Garcia-Payo M.C., Fernandez-Pineda C. (1999). Air gap membrane distillation of sucrose aqueous solutions. J. Membr. Sci..

[29] Dehesa-Carrasco U., Pérez-Rábago C.A., Arancibia-Bulnes C.A. (2013). Experimental evaluation and modeling of internal temperatures in an air gap membrane distillation unit. Desalination.

[30] Shah R.K., London A.L. (1978). Laminar Flow Forced Convection in Ducts: A Source Book for Compact Heat Exchanger Analytical Data. Advances in Heat Transfer.

[31] Khayet M. (2011). Membranes and theoretical modeling of membrane distillation: A review. Adv. Colloid Interface Sci..

[32] Bird R.B., Stewart W.E., Lightfoot E.N. (1976). Transport Phenomena.

[33] Woods J., Pellegrino J., Kozubal E., Slayzak S., Burch J. (2009). Modeling of a membrane-based absorption heat pump. J. Membr. Sci..

[34] Chaudhari S.K., Patel K.R. (2002). Thermodynamic Properties of Aqueous Solutions of Lithium Chloride. Phys. Chem. Liquids.

[35] Wimby J.M., Berntsson T.S. (1994). Viscosity and density of aqueous solutions of LiBr, LiCl, ZnBr_2_, CaCl_2_, and LiNO_3_. 1. Single salt solutions. J. Chem. Eng. Data.

[36] Zhai C., Wu W. (2022). Experimental evaluation on heat/mass transfer and pressure drop of a microchannel membrane-based desorber for compact and efficient H_2_O/LiBr absorption refrigeration. Int. J. Heat Mass Tranf..

[37] de Vega M., Venegas M., García-Hernando N. (2022). Viability on the desorption and air condensation of water in a compact membrane-based microchannel desorber-condenser for cooling applications. Energy Convers. Manag..

[38] He J., Hirata R., Hihara E., Dang C. (2021). Desorption characteristic of LiBr–H_2_O solution in hydrophobic hollow fiber membrane for absorption chiller. Appl. Therm. Eng..

[39] Venegas M., García-Hernando N., De Vega M. (2020). Experimental evaluation of a membrane-based microchannel desorber operating at low desorption temperatures. Appl. Therm. Eng..

[40] Ibarra-Bahena J., Venegas-Reyes E., Galindo-Luna Y.R., Rivera W., Romero R.J., Rodríguez-Martínez A., Dehesa-Carrasco U. (2020). Feasibility Analysis of a Membrane Desorber Powered by Thermal Solar Energy for Absorption Cooling Systems. Appl. Sci..

[41] Hong S.J., Hihara E., Dang C. (2018). Analysis of adiabatic heat and mass transfer of microporous hydrophobic hollow fiber membrane-based generator in vapor absorption refrigeration system. J. Membr. Sci..

[42] Isfahani R.N., Fazeli A., Bigham S., Moghaddam S. (2014). Physics of lithium bromide (LiBr) solution dewatering through vapor venting membranes. Int. J. Multiph. Flow.

[43] Bigham S., Isfahani R.N., Moghaddam S. (2014). Direct molecular diffusion and micro-mixing for rapid dewatering of LiBr solution. Appl. Therm. Eng..

[44] Wang Z., Gu Z., Feng S., Li Y. (2009). Application of vacuum membrane distillation to lithium bromide absorption refrigeration system. Int. J. Refrig..

[45] Sudoh M., Takuwa K., Iizuka H., Nagamatsuy K. (1997). Effects of thermal and concentration boundary layers on vapour permeation in membrane distillation of aqueous lithium bromide solution. J. Membr. Sci..

[46] Ibarra-Bahena J., Rivera W., Romero R.J., Montiel-González M., Dehesa-Carrasco U. (2018). Novel intermittent absorption cooling system based on membrane separation process. Appl. Therm. Eng..

